# Draft Genome Sequences of Local Clinical Isolates of Drug-Resistant and Drug-Sensitive Mycobacterium tuberculosis

**DOI:** 10.1128/MRA.00352-21

**Published:** 2021-06-24

**Authors:** Norzuliana Zainal Abidin, Mohd Nur Fakhruzzaman Noorizhab, Lay Kek Teh, Wai Feng Lim, Noorliza Mohd Noordin, Zirwatul Adilah Aziz, Zamzurina Abu Bakar, Ahmad Izuanuddin Ismail, Norazmi Mohd Nor, Mohd Zaki Salleh

**Affiliations:** a Integrative Pharmacogenomics Institute, Universiti Teknologi MARA Selangor Branch, Selangor, Malaysia; b Faculty of Pharmacy, Universiti Teknologi MARA Selangor Branch, Selangor, Malaysia; c National Public Health Laboratory, Selangor, Malaysia; d Institute of Respiratory Medicine, Ministry of Health, Kuala Lumpur, Malaysia; e Faculty of Medicine, Universiti Teknologi MARA Sungai Buloh Branch, Selangor, Malaysia; f School of Health Sciences, Health Campus, Universiti Sains Malaysia, Kelantan, Malaysia; University of Maryland School of Medicine

## Abstract

In the battle against tuberculosis (TB), plasticity of the Mycobacterium tuberculosis genome is believed to contribute to the pathogen’s virulence and drug resistance. Here, we report 10 draft genome sequences of clinical M. tuberculosis isolated in Malaysia as the basis for understanding the genome plasticity of the M. tuberculosis isolates.

## ANNOUNCEMENT

Mycobacterium tuberculosis is a member of the family *Mycobacteriaceae* and is the causative agent for tuberculosis (TB). Challenges in TB treatment and prevention to curb the spread of TB became complicated due to drug-resistant TB (DR-TB). According to the 2019 Global TB report released by the World Health Organization (WHO), the prevalence rate of TB in Malaysia is at 92 per 100,000 people, with an estimation of 1.2% for multidrug resistant/rifampicin-resistant TB (MDR/RR-TB) (1).

Here, we report five drug-resistant and five drug-susceptible clinical M. tuberculosis isolates collected from pulmonary TB patients at the local hospital in Malaysia before they started on anti-TB treatment. Sputum specimens were collected by clinicians at the respective hospitals and sent to the National Public Health Laboratory (NPHL) for culture and isolation of pure M. tuberculosis; isolates were subsequently tested for their sensitivity and resistance toward anti-TB drugs. Pure M. tuberculosis isolates were cultured on Lowenstein-Jensen medium (L-J) and incubated at 37°C for 3 to 5 weeks. The phenotypic drug susceptibility testing (Phe-DST) was done using the absolute concentration method or proportion method (Bactec MGIT 960 TB detection system) according to the standard protocols (Becton, Dickinson, Sparks, MD, USA) (2–4). Each M. tuberculosis isolate was first tested for susceptibility against the first-line anti-TB drugs. Then, isolates that were resistant toward isoniazid and rifampicin were further tested against the second-line anti-TB drugs. However, isolates which were sensitive toward first-line anti-TB drugs were not subjected to the Phe-DST of the second-line anti-TB drugs. The isolates were heat deactivated and sent to iPROMISE UiTM for whole-genome sequencing.

The genomic DNA of each M. tuberculosis isolate was extracted using the conventional chloroform extraction method (5). According to the manufacturer’s instructions, DNA libraries of each M. tuberculosis isolate were prepared using the Nextera DNA Flex library preparation kit (Illumina, Inc., San Diego, CA) and sequenced using a next-generation sequencer (Illumina, Inc., San Diego, CA). Adaptor sequences and low-quality reads of the paired-end read sequences were trimmed using the Cutadapt tool version 3.4 with Phyton version 2.7.12 (6). The quality of the trimmed reads was assessed using the FastQC software version 0.11.3 with the default parameters (Abraham Bioinformatics, Cambridge, UK). On average, more than 80% of the trimmed reads have Phred scores of >30, with an average coverage depth of 81× and a G+C content of 65%.

An average of more than 99% genome completion was achieved from the *de novo* assembly of the M. tuberculosis genome sequences done using the VelvetOptimizer tool version 1.2.10 (7), and the contigs were scaffolded using the multidraft-based scaffolder (MeDuSa) version 1.6 that mapped the query sequences against reference genome M. tuberculosis H37Rv strain (accession number NC_018143) (8). Variants of the M. tuberculosis genomes were called using the Snippy tool released under the GPL (version 2) (https://github.com/tseemann/snippy). Table 1 shows the details of the assembled M. tuberculosis draft genome sequences and the total number of variants called per genome. In this study, default parameters were used for all the online Web-based tools. M. tuberculosis isolates were classified into different M. tuberculosis lineages based on the octal code of the M. tuberculosis spoligotype patterns using the SpoTyping tool version 2.0 (9) (Table 1).

**TABLE 1:** Details of the assembled M. tuberculosis draft genome sequences

	Accession no. by database											
Isolate^*a*^	NCBI	SRA	BioSample	Total no. of reads	Length (bp [%])^*b*^	No. of scaffolds	*N*_50_ value^*c*^ (bp)	G+C (%)	Cov.^*d*^ (×)	Total no. of variants	Lineage^*e*^	Octal code of M. tuberculosis spoligotype patterns
MR0031	JADPQQ000000000	SRR14270082	SAMN16624767	2,662,964	4,392,022 (99.65)	2	4,389,866	65	79	2,067	L1	774377777413011
MR0021	JADPQR000000000.1	SRR14270083	SAMN16624766	2,792,316	4,375,917 (99.71)	4	4,373,967	65	84	1,443	L2	000000000003771
MR2407	JADPQP000000000	SRR14270081	SAMN16624768	2,787,620	4,380,639 (99.73)	6	4,377,722	65	83	1,425	L2	000000000003771
MR2972	JADPQO000000000	SRR14270080	SAMN16624769	2,832,572	4,391,115 (99.68)	9	4,384,447	65	85	2,123	L2	700000007403771
MR1481	JADPQN000000000	SRR14270079	SAMN16624770	2,611,384	4,365,058 (99.67)	9	4,362,970	65	78	1,353	L2	000000000003771
MS2854	JADPQM000000000	SRR14270078	SAMN16624771	2,685,229	4,370,561 (99.70)	5	4,369,052	65	80	1,402	L2	000000000003771
MS2947	JADPQL000000000	SRR14270077	SAMN16624772	2,736,312	4,401,789 (99.67)	2	4,392,317	65	81	2,089	L3	777777750003331
MS3002	JADPQK000000000	SRR14270076	SAMN16624773	2,696,572	4,366,050 (99.69)	7	4,364,323	65	81	1,383	L2	000000000003771
MS3207	JADPQJ000000000	SRR14270075	SAMN16624774	2,800,646	4,376,767 (99.68)	4	4,373,296	65	84	819	L2	000000000003771
MS3244	JADPQI000000000	SRR14270074	SAMN16624775	2,750,191	4,361,988 (99.70)	5	4,359,624	65	83	1,397	L2	000000000003771

a MS, isolates sensitive toward the first-line anti-TB drugs; MR, isolates resistant toward any of the anti-TB drugs tested.

b Percentage of identity when mapped against the reference genome calculated using.

c *N*_50_ value, sequence length of the shortest contigs at 50% of the total genome length.

d Cov., depth of coverage.

e L1, lineage 1, East African-Indian strain; L2, lineage 2, Beijing strain; L3, lineage 3, Central Asian/Delhi strain.

### Data availability.

Draft genome sequences of the local clinical M. tuberculosis isolates were deposited in the National Center of Biotechnology Information (NCBI) with BioProject number PRJNA674940. The accession numbers of each M. tuberculosis complex (MTBC) genome are included in Table 1.
